# Contribution of Mitophagy to Cell‐Mediated Mineralization: Revisiting a 50‐Year‐Old Conundrum

**DOI:** 10.1002/advs.201800873

**Published:** 2018-07-29

**Authors:** Dan‐dan Pei, Jin‐long Sun, Chun‐hui Zhu, Fu‐cong Tian, Kai Jiao, Matthew R. Anderson, Cynthia Yiu, Cui Huang, Chang‐xiong Jin, Brian E. Bergeron, Ji‐hua Chen, Franklin R. Tay, Li‐na Niu

**Affiliations:** ^1^ Key Laboratory of Shaanxi Province for Craniofacial Precision Medicine Research Department of Prosthodontics College of Stomatology Xi'an Jiaotong University Xi'an 710004 P. R. China; ^2^ State Key Laboratory of Military Stomatology National Clinical Research Center for Oral Diseases Shaanxi Key Laboratory of Stomatology Department of Prosthodontics School of Stomatology The Fourth Military Medical University Xi'an 710032 P. R. China; ^3^ Department of Endodontics The Dental College of Georgia Augusta University Augusta GA 30912 USA; ^4^ Paediatric Dentistry Unit of the Faculty of Dentistry Prince Philip Dental Hospital University of Hong Kong Hong Kong SAR 999077 P. R. China; ^5^ Department of Prosthodontics School and Hospital of Stomatology Wuhan University Wuhan 430079 P. R. China

**Keywords:** amorphous calcium phosphate, mineralization, mitophagy, osteogenesis

## Abstract

Biomineralization in vertebrates is initiated via amorphous calcium phosphate (ACP) precursors. These precursors infiltrate the extracellular collagen matrix where they undergo phase transformation into intrafibrillar carbonated apatite. Although it is well established that ACP precursors are released from intracellular vesicles through exocytosis, an unsolved enigma in this cell‐mediated mineralization process is how ACP precursors, initially produced in the mitochondria, are translocated to the intracellular vesicles. The present study proposes that mitophagy provides the mechanism for transfer of ACP precursors from the dysfunctioned mitochondria to autophagosomes, which, upon fusion with lysosomes, become autolysosomes where the mitochondrial ACP precursors coalesce to form larger intravesicular granules, prior to their release into the extracellular matrix. Apart from endowing the mitochondria with the function of ACP delivery through mitophagy, the present results indicate that mitophagy, triggered upon intramitochondrial ACP accumulation in osteogenic lineage‐committed mesenchymal stem cells, participates in the biomineralization process through the BMP/Smad signaling pathway.

## 1 Introduction

Biomineralization in vertebrates is a ubiquitous and tightly regulated process. Abnormality in this process results in highly morbid pathological conditions such as osteoporosis, osteoarthritis, osteogenesis imperfecta, or Paget's disease. Biomineralization is initiated via amorphous calcium phosphate (ACP) precursors that infiltrate the extracellular collagen matrix and subsequently transform into intrafibrillar carbonated apatite.^1^ The ACP precursor phase, in turn, is produced by coalescence of prenucleation clusters of hydrated calcium and phosphate ionic aggregates.^2^ Although the mechanisms of intrafibrillar mineralization of collagen fibrils have been well established,^1^ the process by which ACP precursors are produced and transported to the extracellular mineralization sites has not been completely elaborated.

Mineral granules have been identified in the intracellular vesicles of osteoblasts; these granules are produced intracellularly and released subsequently by exocytosis to the extracellular environment, as demonstrated by previous studies.^3^ The existence of intracellular calcium phosphate‐containing vesicles in vivo has been validated in zebra fish fin rays[[qv: 3a,4]] and developing mouse bone cells.^5^ These findings indicate that mineral precursors are produced intracellularly and subsequently exported to the extracellular matrix (ECM) to initiate collagen mineralization.

Seminal work conducted on calcium transport in mitochondria identified electron‐dense calcium phosphate granules within the mitochondria when the extracted organelles were exposed to calcium phosphate solutions.^6^ As early as 50 years ago, Lehninger hypothesized that intramitochondrial calcium phosphate deposits in calcifying tissues are released from the mitochondria and transported as stable aggregates to other sites, where they serve as precursors for extracellular mineralization.[[qv: 6a]] However, the biological functions of these intramitochondrial granules have never been deciphered.

Recently, Boonrungsiman et al.^7^ reported that mineral granules are present both within the mitochondria and intracellular vesicles. The authors opined that the process by which mineral granules are transferred from the mitochondria to the intracellular vesicles in osteogenic cells may involve a simple process such as diffusion. Here, we propose that the previously reported, undefined intracellular vesicles containing calcium phosphate granules are autolysosomes, and that the mechanism for transporting mineralization precursors from the mitochondria to the intracellular vesicles involves mitochondrial autophagy, also known as mitophagy, which is a critical process for cell‐mediated biomineralization.

Autophagy is a catabolic process in which cytosolic organelles are sequestered and degraded by lysosomes for recycling into basic components.^8^ Mitophagy is a specific form of autophagy through which damaged or effete mitochondria are delivered to the lysosomes for hydrolytic and enzymatic degradation.^9^ Similar to autophagy, sequestration of mitochondria involves participation of autophagosomes or direct involvement of lysosomal membranes.^9^, ^10^ Using knockdown of autophagy‐essential genes in osteoblast‐specific, autophagy‐deficient mice, autophagy was found to be involved in mineralization of osteoblasts and in bone homeostasis.^11^ Nevertheless, the contribution of mitophagy in hard tissue‐forming cells has not been reported.

Unraveling the process of intracellular calcium phosphate production and transport is important in understanding bone formation as well as the mechanisms involved in pathological mineralization. Accordingly, the objective of the present study was to investigate the intracellular mineral granule transfer pathway by testing the hypothesis that mitophagy functions as the delivery pathway for the transfer of calcium and phosphate‐containing granules from the mitochondria to the lysosomes, using human dental pulp stem cells (hDPSCs) as a model of hard tissue‐forming cells. Although bone marrow stromal cells or osteoblasts were previously used for examination of intracellular calcium phosphate vesicles,[[qv: 3b,7]] hDPSCs were used in the present work because of their superb plasticity, being a promising source of stem cells for hard tissue repair in regenerative medicine.^12^

## 2 Results and Discussion

### 2.1 Evolution of Mitochondrial Electron‐Dense Granules after Osteogenic Induction

**Figure 1:**
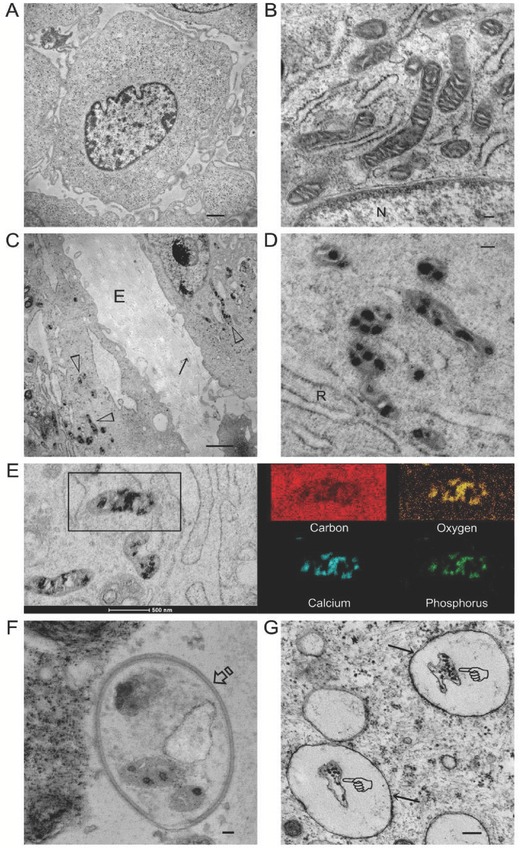
Stained TEM images of intramitochondrial electron‐dense amorphous calcium phosphate (ACP) granules in hDPSCs. A) An undifferentiated cell after exposure to osteogenic differentiation medium for 10 min (bar: 1 µm). B) High magnification TEM taken of an undifferentiated cell from a different site showing healthy mitochondria with double membrane and granule‐free cristae (bar: 100 nm). N: nucleus. C) Differentiated hDPSCs after exposure to osteogenic differentiation medium (ODM) for 48 h (bar: 200 nm). Electron‐dense granules (open arrowheads) were present within the mitochondria. Unmineralized collagen fibrils (arrow) were present in the extracellular matrix (E). D) High magnification TEM taken from a different site showing intramitochondrial ACP granules within a similar cell illustrated in (C) (bar: 100 nm). Selected‐area electron diffraction (SAED) indicated that the granules were amorphous (not shown). R: Rough endoplasmic reticulum. E) STEM‐EDX elemental mapping of the intramitochondrial granules (bar: 500 nm) indicated that they contained calcium, phosphorus, and oxygen. F) A double‐membrane autophagosome (open arrow) with sequestered granule‐containing mitochondria and unidentifiable, partially degraded cytosolic components (bar: 50 nm). G) Single‐membrane autolysosomes (arrows) containing a partially degraded organelle (pointers) with electron‐dense granules (bar: 200 nm).

When undifferentiated hDPSCs exposed to osteogenic differentiation medium (ODM) were examined by transmission electron microscopy (TEM), mitochondria that approximated the rough endoplasmic reticulum exhibited characteristic double membranes with granule‐free cristae (**Figure** **1**A,B). Extrafibrillar collagen fibrils could be seen as early as 48 h (Figure 1C). The first ultrastructural sign of intracellular calcium phosphate accumulation was the appearance of electron‐dense granules within the mitochondria (Figure 1C,D). Those granules could be found commonly during the osteogenic process of the hDPSCs (Movie S1, Supporting Information). Elemental mapping identified calcium, phosphorus, and oxygen in those granules (Figure 1E). Despite reports of the existence of mitochondrial calcium phosphate (CaP) granules in the mid‐1960s to mid‐1970s,^6^, ^13^ there was a vacuum in the literature with respect to the biological functions of these intramitochondrial CaP deposits. Mitochondria are known as the powerhouse of mammalian cells because the majority of adenosine triphosphate (ATP) is generated through the mitochondrial electron transport chain. Besides its principal role in energy production, mitochondria are the first reported intracellular organelles to be associated with Ca^2+^ handling.^14^ Mitochondrial calcium influx and efflux play a fundamental role in cytosolic calcium homeostasis. This calcium buffering capability is responsible for optimal respiratory chain functioning and calcium signaling.^14^ In the presence of inorganic phosphate ions, calcium ions that are sequestered into the mitochondrial matrix are precipitated as CaP.^13^ In the present study, CaP granule‐containing mitochondria were sequestered by double‐membrane organelles (Figure 1F). Based on their double‐membrane structure and engulfing ability, those organelles were probably autophagosomes. Electron‐dense granules were subsequently identified in partially degraded organelles within single membrane vesicles resembling autolysosomes (Figure 1G).

The transfer process of intramitochondrial granules shown in Figure 1 is characteristic of organelle digestion. Mitophagy occurs at a low, basal level in all cells to orchestrate homeostasis of mitochondria biogenesis and degradation.[[qv: 8b,9]] Mitophagy is also involved in developmental processes that require mitochondrial clearance; impairment of mitophagy drastically alters mitochondrial function and cell fate in many cell types.^15^ Although augmented autophagic flux was identified during osteoblast differentiation and mineral nodule formation,^16^ the basis in which osteogenic differentiation is modulated by autophagy is unclear.

Examination of mitophagy and osteogenesis‐related gene expressions with quantitative reverse‐transcription polymerase chain reaction (qRT‐PCR) identified that mitophagy was maintained at the baseline level in undifferentiated hDPSCs. In contrast, mitophagy‐related genes increased two to five folds after hDPSCs were exposed to ODM for 12 h. Mitophagy continued after 24 and 48 h of ODM exposure and returned to the baseline level after 72 h. In the present study, the ODM was changed every 3 d. From the results of Figure S1 (Supporting Information), mitophagy gene expressions were in close relationship with stimulus derived from fresh ODM; mitophagy‐related genes increased at day 4 and returned to the baseline at day 6 (Figure S1, Supporting Information). Results from the present work reveal, for the first time, that mitophagy, the specific organelle autophagy process, is up‐regulated during osteogenic differentiation of stem cells.

### 2.2 Interaction of Intramitochondrial Granules with Single Membrane Vesicles

Calcium phosphate granules present in cytosolic single membrane vesicles are released by exocytosis to the extracellular matrix to initiate mineralization.^3^ The existence of CaP‐loaded intracellular vesicles had also been reported by Rhode and Mayer in 2007[[qv: 3c]] and more recently by the Weiner group in the early 2010s.^3^ Nevertheless, the interaction between CaP granules within the mitochondria and intracellular vesicles remains obscure.

**Figure 2:**
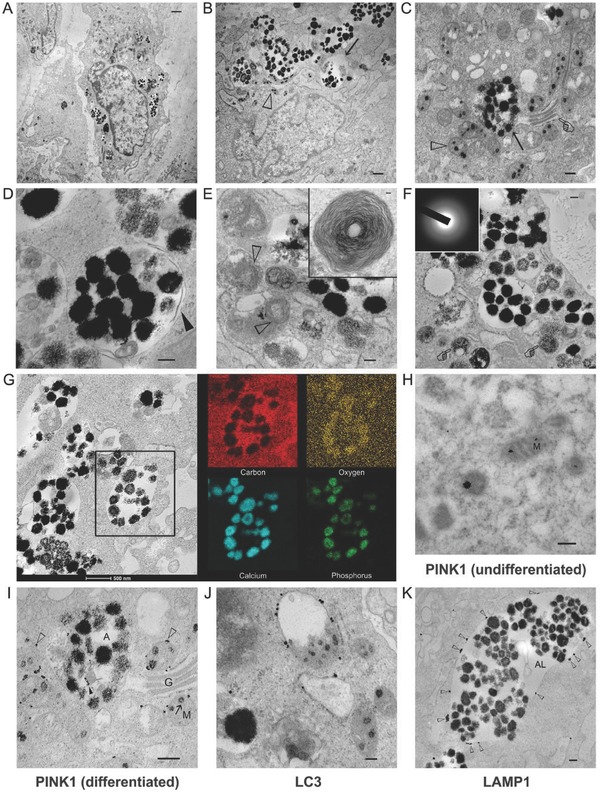
TEM images illustrating the relationship between the electron‐dense granules within mitochondria and those within the cytosolic vesicles of hDPSCs. A) hDPSCs after exposure to ODM for 2 weeks (bar: 1 µm). B) High magnification of the intramitochondrial ACP granules (open arrowhead) and intravesicular granules (arrow) (bar: 50 nm). C) Coexistence of intramitochondrial ACP granules (open arrowhead), autolysosome (arrow), and Golgi apparatus (pointer) (bar: 200 nm). D) An autolysosome containing electron‐dense granules and other unidentifiable cytosolic components (bar: 200 nm). Partial degradation of the inner membrane derived from the original autophagosome could be seen (arrowhead). E) Multilamellar bodies identified (open arrowheads) adjacent to autolysosomes (bar: 200 nm). The inset shows a multilamellar body in higher magnification (bar: 50 nm). F) Granule‐containing autolysosomes in the vicinity of lysosomes (pointer) (bar: 200 nm). The electron‐dense granules were amorphous (inset). The granules were very close to the extracellular matrix. G) STEM‐EDX elemental mapping of the intravesicular electron‐dense granules revealed the presence of calcium, phosphorus, and oxygen, suggesting that those granules were ACP. H–K) Immunogold labeling of hDPSCs (bars: 200 nm for all images). H) In undifferentiated mitochondria (M) without granules, PINK1 was located within the interior of the mitochondria. I) In mitochondria destined for degradation, PINK1 was predominantly located (open arrowheads) along the periphery of the mitochondria (M). Some PINK1 could be identified around degraded organelles (solid arrowhead) within the autolysosome (A). G: Golgi body. J) LC3 was predominantly located along the membrane of mitochondria‐containing autophagosomes. K) LAMP1 was predominantly located along the membrane (open arrowheads) of the granule‐containing autolysosomes (AL).

After 2 weeks of osteogenic induction, electron‐dense granules were concomitantly identified by TEM in the mitochondria and within single membrane cytosolic vesicles (**Figure** **2**A,B). Within the same differentiated hDPSC, granules in the vesicles were considerably larger than those present in the mitochondria (Figure 2C). This may be explained by the re‐aggregation of ACP in the vesicles due to higher concentration of calcium and phosphate after transfer of those granules from different mitochondria. Stevens and her colleagues subsequently provided evidence of mitochondria–vesicle interactions and speculated that calcium and phosphate were transferred from the mitochondria to intracellular vesicles via diffusion.^7^ However, the exact manner of communication and the biological implication of such an interaction had not been elucidated.

Because intravesicular organelle components and partially degraded inner membranes were found within the cytosolic vesicles, those entities are likely to be autolysosomes (Figure 2D). Potential involvement of mitochondria autophagy is further supported by the observation of abundant multilamellar bodies adjacent to the cytosolic vesicles (Figure 2E); these bodies have been reported to be late lysosomal organelles or autolysosomes generated by active autophagic flux.^17^

Intravesicular granules were amorphous (Figure 2F) and had the same elemental composition as the intramitochondrial granules (Figure 2G), indicating that they contained ACP. Larger ACP granules were identified in the autolysosomes, probably because of the aggregation of fluidic ACP droplets derived from the degraded mitochondria. These droplets are liquid‐like and moldable prior to their transformation into crystalline CaP phases.^1^, ^18^ After their transfer to the extracellular milieu via exocytosis,^3^ the ACP droplets remained amorphous initially (Figure S2A, Supporting Information) and co‐existed with the unmineralized collagen fibrils (Figure S2B, Supporting Information). Infiltration of ACP into collagen fibrils resulted in their intrafibrillar mineralization (Figure S2C, Supporting Information). In a cell‐culture environment, transformation of ACP into apatite crystallites also resulted in the formation of mineral nodules within the extracellular matrix (Figure S2D, Supporting Information).

Phosphatase‐and‐tensin homolog‐induced putative kinase 1 (PINK1) is a key regulator for initiating mitophagy in mammalian cells.^19^ PINK1 is degraded rapidly and maintained at very low level in normal mitochondria. However, PINK1 rapidly accumulates on mitochondrial membranes once the mitochondria are damaged.^19^ Accumulation of PINK1 sends a “eat‐me” signal that induces translocation of *PARKIN* from the cytosol to dysfunctioned mitochondria and targets the mitochondria for lysosomal degradation.^19^, ^20^ Accordingly, immunogold labeling of PINK1, lysosomal‐associated membrane protein 1 (LAMP1, lysosome marker^21^), and microtubule‐associated protein‐1 light chain‐3 (LC3, autophagosome marker^22^) was performed to confirm the identity of the organelles previously identified by TEM. In mitochondria derived from undifferentiated hDPSCs, granules were absent and PINK1 was located inside the mitochondria (Figure 2H). After osteogenic differentiation, PINK1 accumulated predominantly along the periphery of the granule‐containing mitochondria, indicating those mitochondria were dysfunctioned (Figure 2I). The use of anti‐LC3 antibodies confirmed that autolysosomes were involved in intracellular CaP transport (Figure 2J). Immunolabeling of LAPM1 proteins on the surface of the cytosolic vesicles confirmed that these entities were autolysosomes (Figure 2K). The immuno‐TEM data suggested the possibility of the involvement of mitophagy‐associated organelles in intracellular transportation of ACP from the mitochondria to autolysosomes.

The mitochondrial electron transport chain translocates hydronium ions across the inner mitochondrial membrane and generates mitochondria membrane potential (Δ*Ψ*_m_).^22^ Maintaining normal Δ*Ψ*_m_ is critical for mitochondrial functions.^14^, ^22^ As mentioned previously, liquid‐like ACP precursors accumulated within the mitochondria during osteogenic differentiation of hDPSCs. Concomitantly, increasing amount of matrix Ca^2+^ triggers mitophagy of the mitochondria.^23^ These organelles respond to stressful stimuli such as Ca^2+^ overloading in a hierarchical manner such as mitophagy, apoptosis, or necrosis.^23^, ^24^ In the present context, it appears that mitophagy is the most logical strategy for maintaining hDPSCs in a healthy, viable state during osteogenesis. From the TEM images (Figure 2A,B), it is apparent that cell nuclei were intact and no cell blebbing or apoptotic body was identified in the osteogenic lineage‐committed hDPSCs and those undergoing active osteogenesis.

### 2.3 Effects of Mitophagy Inducer and Inhibitor on Osteogenesis

The mitophagy inducer carbonyl cyanide 3‐chlorophenylhydrazone (CCCP) and the inhibitors bafilomycin A1 (Baf‐A1) and cyclosporin A (CsA) were used as positive and negative controls, respectively. Different from the other studies that investigated the short‐term effects of mitophagy, lower concentrations of CCCP (500 × 10^−9^ m), Baf‐A1 (10 × 10^−9^ m), and CsA (50 × 10^−9^ m) were used as the respective optimized concentration for evaluating their effects on osteogenesis of hDPSCs. The use of reagents at these concentrations did not result in significant toxicity (Figure S3A, Supporting Information) or apoptosis (Figure S3B, Supporting Information) of the hDPSCs. The effectiveness of these agents as mitophagy stimulator and inhibitors was confirmed by confocal laser scanning microscopy (Figure S4, Supporting Information) and mitochondria membrane potential (Δ*Ψ*_m)_ evaluation (Figure S5, Supporting Information).

**Figure 3:**
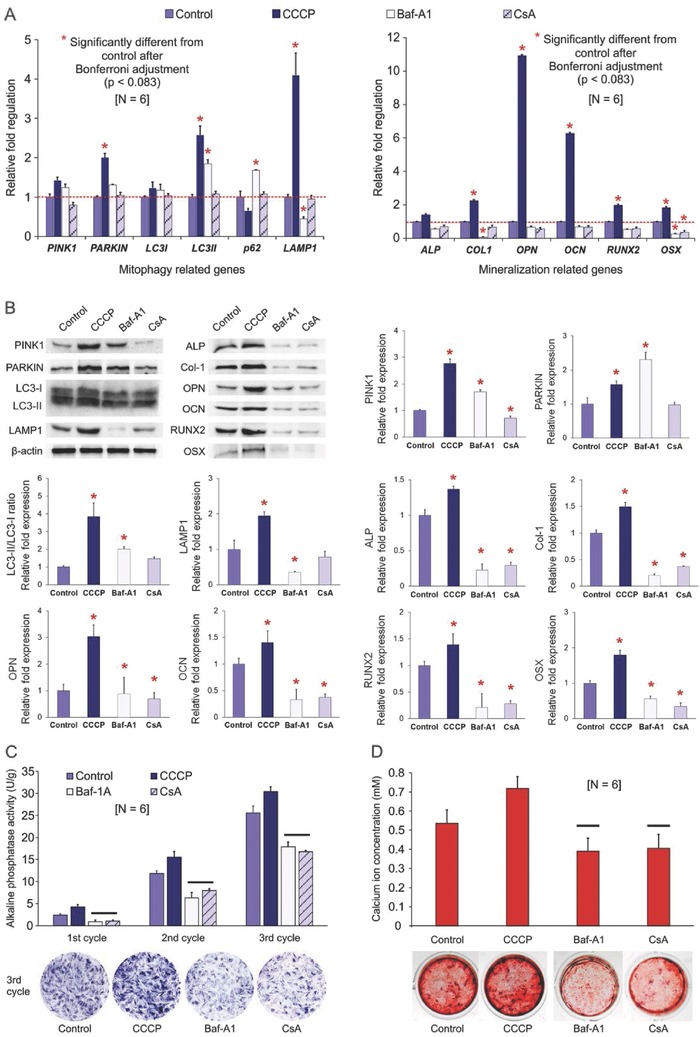
Osteogenesis evaluation of differentiated hDPSCs treated with mitophagy stimulator and inhibitors. A) qRT‐PCR of differentiated hDPSCs (control) and hDPSCs treated with CCCP, Baf‐A1, or CsA for “one chemical reagent administration cycle” (see text). Left: Changes in fold regulations of mitophagy‐related genes. For each gene, group columns identified with asterisks were significantly different from the control (*p* < 0.0071 after Bonferroni adjustment). Right: Changes in fold regulations of mineralization‐related genes. For each gene, group columns identified with asterisks were significantly different from the control (*p* < 0.00083 after Bonferroni adjustment). B) Western blot results of selected mitophagy‐related and mineralization‐related proteins obtained from differentiated hDPSCs (control) and hDPSCs treated with CCCP, Baf‐A1, or CsA for three “chemical reagent administration cycles”. For each protein, group columns identified with asterisks were significantly different from the control (*p* < 0.05). C) ALP enzyme activity of the four groups after one to three “chemical reagent administration cycles.” For each cycle, group columns connected with a bar were not significantly different (*p* > 0.05). Representative ALP staining of the hDPSCs after three cycles is shown at the bottom of the chart. D) Analysis of extracellular calcium content in the four groups after four “chemical reagent administration cycles,” Group columns labeled with bars were not significantly different (*p* > 0.05). Representative alizarin red S‐stained hDPSCs are shown at the bottom of the chart.

Being the most commonly inducer of mitophagy in mammalian cells, CCCP exerts its effects via Δ*Ψ*_m_ reduction.^25^, ^26^ PINK1 rapidly accumulates on the mitochondrial outer membrane when Δ*Ψ*_m_ is reduced, resulting in sequestration of damaged mitochondria by autophagosomes.^19^ LC3 is a specific marker for autophagosomes[[qv: 21b]] and consists of three members (LC3A, LC3B, and LC3C) in humans.^27^ During autophagosome formation, the cytosolic form of LC3 (LC3‐I) is lipidated, producing LC3‐II that binds tightly to autophagosomes.^21^ In the presence of CCCP, most of the mitophagy‐related genes (*PARKIN, LC3B, LAMP1, LAMP2*) were up‐regulated after 72 h of ODM exposure when compared with control hDPSCs, except for P62 (**Figure** **3**A). This is because decrease in P62 signifies increased autophagy turnover.^28^ Interestingly, the osteogenesis‐related genes (*COL1, OPN, OCN, RUNX2, OSX*) were also up‐regulated simultaneously (Figure 3A). Results of the western blots (Figure 3B) showed similar trends; the expression level of mitophagy‐related proteins PINK1, PARKIN, the ratio of LC3II/LC3I, LAMP1, and the osteogenesis‐related proteins ALP, COL1, OPN, OCN, RUNX2, and OSX were all up‐regulated. Thus, CCCP at low concentration enhances the osteogenesis potential of hDPSCs. This is supported by increases in ALP activity (Figure 3C) and extracellular calcium deposition (Figure 3D). Concomitant augmentation of mitophagy and osteogenesis is indicative of the contribution of mitophagy to osteogenic differentiation and mineralization of hDPSCs.

Mitophagy is reliant upon fusion of mitochondria‐containing autophagosomes with lysosomes for digestion of the sequestered cargo. Bafilomycin A1 is a potent inhibitor of vacuolar H^+^ATPase, which controls pH in the lysosomes (V‐ATPase).^29^ Hence, bafilomycin inhibits autophagic flux by preventing acidification of lysosomes. Because the last step of mitophagy was impaired by the V‐ATPase inhibitor bafilomycin, as shown by decreased LAMP1 in both PCR and western blot results (Figure 3A,B), dysfunctioned mitochondria could not be degraded by lysosomes. This was demonstrated concomitantly by western blotting. Increase in protein levels of PINK1 and PARKIN, and the ratio of LC3II/LC3I are attributed due to the accumulation of autophagosomes (Figure 3B).

Whereas bafilomycin blocks the latter stage of mitophagy/autophagy, CsA, an inhibitor of mitochondrial permeability transition pore, inhibits the early stage of mitophagy.^30^ When CsA was used simultaneously with other stimuli that activate mitophagy, such as starvation^31^ or chemical reagents,^30^, ^32^ inhibition of mitophagy was effectuated via stabilization of mitochondrial permeability transition. When CsA was used alone in the present study, it has no significant effect on mitophagy‐related genes except for PINK1 (Figure 3B), due to reduced accumulation of PINK1 on the mitochondria. Both mitophagy and autophagy involve the participation of LC3, P62, and LAMP1. Inhibition of mitophagy at its early stage by CsA did not block autophagy; this was demonstrated by the results presented in Figure 3B. CsA reduced osteogenesis of hDPSCs significantly, which may be explained by the inhibition effect of calcineurin by CsA. It has been shown that CsA causes profound bone loss in humans and in animal models. This is because calcineurin regulates bone formation through inhibition of osteoblast differentiation.^33^ Therefore, CsA may affect osteogenesis via another mechanism that is independent of mitophagy.

Autophagosomes accumulate either by inducing autophagy (CCCP) or by blocking autophagic flux at the latter stage of mitophagy (Baf‐A1). The latter occurs because autophagosomes that normally fuse with lysosomes to form autolysosomes do not breakdown and gradually accumulate in the cytosol. This is the rationale for using two mitophagy inhibitors for blocking mitophagy at different stages. Osteogenesis is impaired as long as mitophagy was intercepted by Baf‐A1 or CsA, as demonstrated by the reductions in osteogenesis‐related proteins (Figure 3B) and osteogenic potential of the hDPSCs (Figure 3C,D).

### 2.4 PINK1 Knockdown Impairs Osteogenesis via the Bone Morphogenetic Protein/Small‐Mothers‐Against‐Decapentaplegic Signaling Pathway

Lentiviral vectors derived from the human immunodeficiency virus have become major tools for gene delivery in mammalian cells. The advantage of using lentivirus vectors is their potency in mediating transduction and stable expression in dividing and nondividing cells, both in vitro and in vivo. In the present study, a lentivirus vector incorporating PINK1‐targeted small interfering ribonucleic acid (siRNA) was used to knockdown PINK1 expression in hDPSCs, to definitely confirm the role of mitophagy in osteogenesis, and to evaluate the associated mechanism. Lentivirus transfection efficiency was examined with fluorescence microscopy and further evaluated by flow cytometry. More than 90% of the transfected hDPSCs were green fluorescent protein‐positive, compared with 1.8% in the nontransfected control (Figure S6A,B, Supporting Information). Concomitantly, PINK1 expression was significantly reduced, as demonstrated by PCR (*p* < 0.001) and western blot after PINK1 knockdown (Figure S6C,D, Supporting Information).

**Figure 4:**
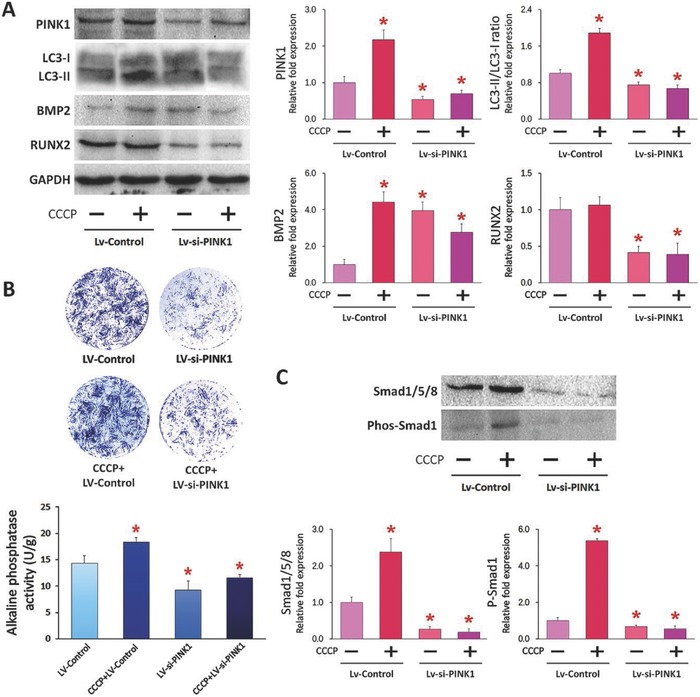
ALP activity and protein expression level of PINK1‐silenced hDPSCs. A) Western blot results of selected mitophagy‐related and mineralization‐related proteins obtained from PINK1‐silenced hDPSCs treated with or without CCCP. B) Results of ALP staining and ALP enzyme activity. C) Western blot results of Smad1/5/8 and phosphate Smad1 expression. For each target, group columns identified with red asterisks were significantly different from the control group (Lv‐Control, *p* < 0.05).

Western blot was performed to investigate the relative protein changes in PINK1 silencing‐induced mitophagy and osteogenesis (**Figure** **4**A). PINK1 expression increased significantly in the LV‐Control group (empty lentiviral vector) treated with CCCP. In contrast, PINK1 expression was significantly reduced in the LV‐si‐PINK1 group (group with silenced PINK1) treated with CCCP. There was no significant difference in PINK1 expression between the LV‐si‐PINK1 groups treated with or without CCCP. PARKIN expression was decreased after PINK1 silencing (Figure S7, Supporting Information). The LC3 II/LC3 I ratio was also decreased in both LV‐si‐PINK1 groups with or without CCCP treatment (Figure 4A). These results are indicative of suppressed mitophagy after PINK1 silencing.

Previous studies have reported in depth the molecular mechanism associated with osteogenesis and bone regeneration.^34^ Among the factors involved, the bone morphogenetic protein/Small‐mothers‐against‐decapentaplegic (BMP/Smad) signaling pathway plays a key role in osteoblast differentiation of mesenchymal stem cells. In particular, BMP‐regulated Smad is crucial during the initial stage of osteogenesis.^35^ In the present study, treatment with CCCP enhanced BMP2 expression in both the LV‐Control and LV‐si‐PINK1 groups (Figure 4A); BMP2 expression was also increased in the LV‐si‐PINK1 group without CCCP treatment. In both LV‐si‐PINK1 groups treated with or without CCCP, RUNX2 expression was reduced compared with the empty vector LV‐Control group. There was no significant difference in BMP4 and Smad4 expressions between LV‐Control and LV‐si‐PINK1 while OSX expression was significantly decreased in the LV‐si‐PINK1 group (Figure S7, Supporting Information). Quantitative analysis of ALP staining after treatment with the lentiviral vector LV‐si‐PINK1 for 7 d indicated significant reduction in intracellular ALP activity (Figure 4B). Previous studies demonstrated that expression of the transcription factor Runx2/Cbfa1, which initiates and regulates osteogenic differentiation of mesenchymal stem cells, is controlled by BMP‐activated Smads.^36^ Enhanced BMP2 expression is directly mediated by the osteogenic differentiation medium [[qv: 35a]] and RUNX2 is positively regulated by the BMP/Smad signaling pathway.^35^ In the present work, RUNX2 expression was decreased in the LV‐si‐PINK1 group, which is vital during the initial stage of osteogenesis. In summary, expression of BMP2 was increased after osteogenic induction while Runx2 expression and osteogenesis of hDPCSs were not enhanced after PINK1 silencing. Taken together, PINK1 silencing of hDPSCs causes disruption of the BMP‐Smad‐Runx2 transduction pathway.

Expression of Smad1/5/8 and activation of Smad1 were also related to the extent of mitophagy (Figure 4C). In the LV‐Control group with CCCP treatment, Smad1 expression was significantly increased when compared with the LV‐Control. After PINK1 silencing, Smad1/5/8 and Smad1 activities were significantly decreased regardless of CCCP treatment. Blockage of the BMP‐Smad linkage and reduction in Smad1/5/8 expression after PINK1 silencing strongly suggest that decreased BMP‐Smad signaling is involved when osteogenesis is compromised in mitophagy‐impaired hDPSCs.

Recent studies provide clues on the involvement of BMP/Smad signaling in mitophagy. The ubiquitin ligase Smurf1 has been shown to participate in mitophagy mediation.^37^ Murine embryonic fibroblasts lacking Smurf1 are incapable of autophagosomal targeting of Sindbis and herpes simplex viruses and in the clearance of damaged mitochondria.[[qv: 37b]] After binding of Smurf1 to the WW domain, Smad1 and Smad5 are ubiquitinated and degraded in the proteasome.^38^ Smurf1 controls BMP signaling by maintaining steady‐state protein levels of Smad1 and Smad5.^38^, ^39^ Although Smurf1‐related mitophagy is responsible for the inhibition of osteogenesis, the exact function of Smurf1 in mitophagy is still unknown.[[qv: 37a,40]] Bone morphogenetic protein exerts its diverse biological effects through the transmembrane receptors BMPR‐I and BMPR‐II.[[qv: 35a]] The latter has been shown to be responsible for recruiting and phosphorylating R‐Smads.^41^ Autophagy/mitophagy‐induced lysosomal acidification causes BMPR‐II degradation,^42^ which, in turn, inhibits Smad activation and BMP/Smad‐regulated osteogenesis. In addition, the ERK1/2 transduction pathway has also been reported to be an essential factor in up‐regulating Smad1/5/8 expression.^41^, ^43^ Detailed investigations of the signaling pathways involved in mitophagy are beyond the scope of the present work. Further studies are required to fully comprehend the molecular mechanisms involved in the contribution of mitophagy to BMP/Smad‐regulated osteogenesis.

## 3 Conclusions

**Figure 5:**
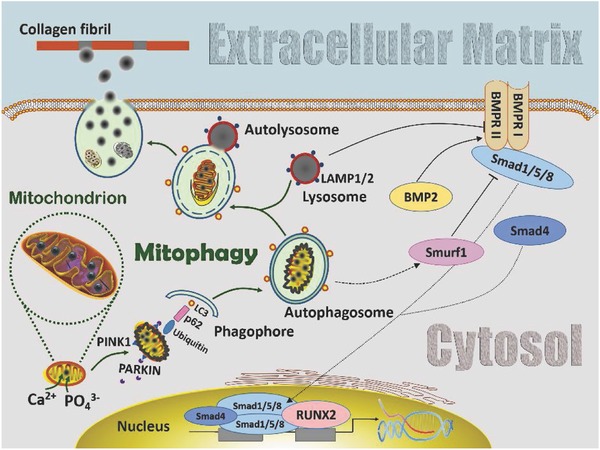
Schematic of involvement of mitophagy in the transportation of amorphous calcium phosphate (ACP) precursors from the mitochondria to the extracellular matrix in stem cells with osteogenic potential. Calcium and phosphate ions are sequestered into the mitochondria matrices and accumulate as ACP granules. When ACP accumulation reaches a threshold, the mitochondria swell and lose membrane potential, resulting in PINK1 accumulation on their outer membrane. The latter recruits PARKIN from the cytosol to the damaged mitochondria. PARKIN ubiquitylates mitochondrial proteins and causes the ACP‐overloaded mitochondria to be engulfed by autophagosomes through p62 and LC3. The autophagosomes then fuse with lysosomes to become autolysosomes. Digestion of the dysfunctioned mitochondria by autolysosomal acidification releases the intramitochondrial ACP droplets, which coalesce into larger ACP granules within the autolysosomes. The coalesced ACP granules are subsequently transferred from the autolysosomes to the ECM via exocytosis. Infiltration of ACP into collagen fibrils results in intrafibrillar mineralization of the extracellular matrix. During this process, the BMP/Smad signaling pathway is activated to support the ACP transportation role of mitophagy in cell‐mediated osteogenesis. Abbreviations for pathway modification within the cytosol: solid black arrow: direct stimulatory modification; dashed black arrow: tentative stimulatory modification; dotted black arrow: joining of subunits and translocation; solid folding line with arrow: transcriptional stimulatory modification; solid line with right vertical bar: direct inhibitory modification. BMRR I and BMRR II: bone morphogenetic protein receptor I and II.

The present study identified mitophagy as a mechanism for ACP delivery in osteogenic lineage‐committed mesenchymal stem cells. In addition, mitophagy participates in the cell‐mediated biomineralization process through the BMP/Smad signaling pathway. These findings are summarized collectively in **Figure** **5**. When stem cells are exposed to an environment that is conducive toward osteogenesis, calcium ions that are sequestered into the mitochondrial matrix are precipitated as ACP in the presence of inorganic phosphate ions. The accumulated ACP triggers mitophagy to initiate ACP transportation intracellularly between cytosolic organelles. With the help of PINK1/PARKIN, ACP‐containing mitochondria are sequestered by autophagosomes, which, upon fusion with lysosomes, become autolysosomes. Digestion of the dysfunctioned mitochondria by autolysosomal acidification releases the intramitochondrial ACP droplets, which coalesce into larger ACP granules within the autolysosomes. The coalesced ACP granules are subsequently transferred from the autolysosomes to the extracellular matrix via exocytosis. Infiltration of the ACP into the collagen fibrils results in intrafibrillar mineralization of the collagen fibrils within the extracellular matrix, as well as phase transformation of the ACP into apatite‐containing mineral nodules in the biomineralization process.

## 4 Experimental Section

*Cell Culture*: hDPSCs were obtained from 18–25‐year‐old human subjects according to a protocol approved by the Ethics Committee of the Fourth Military Medical University. Cells with CD29^+^/CD44^+^/CD90^+^/CD105^+^/CD146^+^/STRO‐1^+^/CD34^−^/CD45^−^ immunophenotype were previously characterized by flow cytometry; their multipotency was established using chrondrogenic, adipogenic, and osteogenic culture conditions.^44^ The growth medium consisted of Dulbecco's Modified Eagle medium (Thermo Fisher Scientific, Waltham, MA, USA) supplemented with 10% fetal bovine serum, 100 units mL^−1^ penicillin, and 100 mg mL^−1^ streptomycin. Passages 2–4 hDPSCs were cultured to ≈70% confluency prior to replacement with ODM. The latter, consisting of complete growth medium supplemented with 50 mg L^−1^ ascorbic acid, 10 mmol L^−1^ glycerophosphate, and 100 nmol L^−1^ dexamethasone, was changed every 3 d.

*Ultrastructural Examination*: Lineage‐committed hDPSCs were examined by TEM after culturing in ODM for different time periods (up to 3 weeks), to identify intracellular and extracellular mineral deposits. Cells were detached with trypsin, centrifuged, fixed with glutaraldehyde, postfixed with 1% osmium tetroxide, dehydrated in an ascending ethanol series, transferred to propylene oxide, and embedded in epoxy resin. Ninety nanometer‐thick sections were stained with uranyl acetate and Reynold's lead citrate, and examined using a JEM‐1230 TEM (JEOL, Tokyo, Japan) at 110 kV. Besides, a serial of sections were cut by serial section cutting technique, and the acquired images were matched and 3D reconstructed by Amira 6.0.1 software (Thermo Fisher Scientific, USA). Selective area electron diffraction (SAED) was performed on electron‐dense granules in the intracellular vesicles.

Immuno‐TEM was used to identify mitochondrial, lysosomal, and autolysosomal involvement in the osteogenic lineage‐committed hDPSCs.^26^ Cells cultured in ODM for 7 d were embedded with LR‐White resin (Electron Microscopy Sciences, Ft. Washington, PA, USA). Grids were etched with H_2_O_2_ in phosphate‐buffered saline, quenched with ammonium chloride, blocked with bovine serum albumin, and stained with primary antibodies. The latter consisted of mouse monoclonal anti‐PINK1 for dysfunctioned mitochondria (ab75487, 1:250 dilution), rat monoclonal anti‐LAMP1 for autolysosomes (ab25245, 1:250 dilution), and rabbit polyclonal anti‐LC3 for autophagosomes (ab128025, 1:250 dilution) (all from Abcam, Cambridge, MA, USA). The corresponding anti‐species nanogold conjugates (1.4 nm nanogold; Nanoprobes, Yaphank, NY, USA; 1:1500 dilution) were used as secondary antibodies. Grids containing nanogold particles were silver‐enhanced with HQ Silver (Nanoprobes) and examined with the JEM‐1230 TEM.

*Elemental Mapping*: Elemental mapping was performed using a scanning TEM (STEM) equipped with energy dispersive X‐ray spectroscopy (EDX). Specimens used for elemental mapping were not postfixed with osmium tetroxide to avoid interference during EDX. Unstained thin sections were examined with a Technai G2 STEM (FEI, Hillsboro, OR, USA) at 200 kV. Spectrum acquisition and elemental mapping were conducted using an INCA X‐sight detector (Oxford Instruments, Abingdon, United Kingdom). Mappings were acquired with the FEI TIA software using a spot dwell time of 300 ms with drift correction performed after every 30 images.

*Mitophagy Induction/Inhibition*: Established hDPSCs were cultured in growth medium to ≈70% confluency prior to incubation in ODM. The cells were harvested using 0.25% trypsin and seeded in 6‐well plates with equal cell number in each well. All groups were incubated initially in ODM for 1 week. The cells were divided into four groups (CCCP, Baf‐A1, CsA, and control) and treated with the selected concentration of each mitophagy stimulator/inhibitor. Methods and results of determining the optimal concentrations of mitophagy inducer/inhibitors are provided in the Supporting Information. Cells from the three experimental groups were subsequently cultured for 2 d in ODM containing optimized concentration of CCCP, Baf‐A1, or CsA. Cells from the control were cultured in pure ODM only. All groups were cultured in pure ODM for an additional 2 d. This “chemical reagent administration cycle” was adopted to minimize the cytotoxic effects of the mitophagy stimulant/inhibitor on osteogenic differentiation. The cycle was repeated three times during the experimental period.

*Gene Expression*: qRT‐PCR was performed after the use of the mitophagy stimulator/inhibitor for one cycle. The genes included *PINK1*, *PARKIN*, microtubule‐associated protein‐1 light‐chain 3 (*LC3I, LC3II*), ubiquitin‐binding adaptor p62 (*p62*), *LAMP1*, alkaline phosphatase (*ALP*), collagen type I (*COL1*), osteopontin (*OPN*), osteocalcin (*OCN*), runt‐related transcription factor 2 (*RUNX2*), and osterix (*OSX*) (Table S1, Supporting Information). Glyceraldehyde‐3‐phosphate dehydrogenase (*GAPDH*) was used as housekeeping gene. Total RNA from experimental/control hDPSCs was isolated, purified, and reverse‐transcripted using PrimeScript RT reagent kit (Takara Bio Inc., Shiga, Japan). Experiments were performed in sextuplicate (*N* = 6) in a 7500 real‐time PCR System (Applied Biosystems, Carlsbad, CA, USA). Results were calculated using the 2^−ΔΔCt^ method and presented as fold regulations relative to the control.

*Protein Expression*: Western blot was used to examine protein expressions of PINK1, PARKIN, LC3, LAMP1, ALP, COL1, OPN, OCN, RUNX2, and OSX after the use of the mitophagy inducer/inhibitor for three cycles (Section “Experimental groups”). Proteins derived from the lysed cells were loaded on a polyacrylamide gel‐plate for electrophoresis and transferred to a polyvinylidene difluoride membrane. The membrane was blocked with nonfat milk and incubated with anti‐PINK1 (ab23707), anti‐PARKIN (ab15954), anti‐LC3I/II (ab128025m), anti‐LAMP1 (ab24170), anti‐ALP (ab95462), anti‐COL1 (ab138429), anti‐OPN (ab91655), anti‐OCN (ab133612), anti‐RUNX2 (ab23981), and anti‐OSX (ab22552) (all from Abcam). Signals were identified using horseradish peroxidase‐conjugated secondary antibody and enhanced chemiluminescence detection. Protein expression levels were normalized against GAPDH.

*Alkaline Phosphatase Enzyme Activity*: Differentiated hDPSCs exposed to the mitophagy stimulator/inhibitor for one to three cycles (Section “Experimental groups”) were stained with an ALP color development kit (Beyotime, Shanghai, China). Enzyme activity was determined with an ALP detection kit (Genmed, Shanghai, China). Absorbance was measured immediately at 405 nm. Total protein concentration was determined using BCA protein quantification (Beyotime). Activity was normalized against the total protein content and calculated as optical density per mg protein.

*Extracellular Mineralization*: Alizarin red S staining was performed (*N* = 6) after exposure of the hDPSCs to the mitophagy stimulator/inhibitor for three cycles (Section “Experimental groups”). Cells were fixed in paraformaldehyde and stained with alizarin red S (pH 4.2). Stained calcium deposits were dissolved in 10% acetic acid and neutralized with 10% NH_4_OH prior to quantification with a spectrophotometer at 405 nm. Dye concentration was determined from a calibration curve that correlated absorbance with known dye concentrations. Because one mole of dye binds to two moles of calcium to produce an alizarin red S–calcium complex,^45^ calcium deposition was expressed as molar equivalent of calcium.

*Lentivirus Infection*: A human PINK1‐specific siRNA lentivirus vector containing green‐fluorescent protein (GFP) marker (Genechem, Shanghai, China) was used for knockdown of PINK1 and designated as LV‐si‐PINK1. An empty lentiviral vector that expressed GFP only was used as control (LV‐Control). The hDPSCs were transfected with LV‐si‐PINK1 or LV‐Control virus particles (40 particles per cell) in the presence of 5 µg mL^−1^ hexadimethrine bromide (Polybrene, MilliporeSigma, Burlington, MA, USA) to improve transduction efficiency. Cells were examined using fluorescence microscopy and flow cytometry (FACS‐Calibur; Becton Dickinson, Franklin Lakes, NJ, USA) to analyze transfection efficiency. Both qRT‐PCR and western blot were used to determine PINK1 knockdown efficacy.

The transfected hDPSCs were treated with different reagents after demonstrating the effectiveness of LV‐si‐PINK1. The study design comprised four groups, designated, respectively, as [Vector, CCCP‐]: LV‐Control infected cells treated without CCCP (negative control); [Vector, CCCP+]: LV‐Control infected cells treated with CCCP; [Si‐PINK1, CCCP‐]: LV‐si‐PINK1 infected cells treated without CCCP; and [Si‐PINK1, CCCP+]: LV‐si‐PINK1 infected cells treated with CCCP. After the treated cells were cultured in ODM for 2 d, the total protein content from each group was extracted for western blot. Apart from the aforementioned PINK1, RUNX2, PARKIN, and LC3 targets, proteins involved in the BMP/Smad signaling pathway were also analyzed. Anti‐BMP2 (ab214821), anti‐BMP4 (ab124715), anti‐Smad1/5/8 (ab66737), and anti‐phos‐Smad1 (ab214423) were employed for western blot (all from Abcam). Cells cultured for 7 d were used for the examination of ALP activity.

*Statistical Analyses*: Data derived from each parameter were examined for their compliance with the normality and homoscedasticity assumptions required for the use of parametric analytical methods. Data sets that conform to those assumptions were examined with one‐factor analysis of variance and Holm–Šidák pairwise comparison procedures. Noncompliant data were nonlinearly transformed prior to the use of parametric methods. When nonlinear data transformation was incapable of satisfying those assumptions, Kruskal–Wallis analysis of variance and Dunn's test were used for analyses. Statistical significance were set at α = 0.05. For analysis of qRT‐PCR results, Bonferroni correction of the alpha value was performed for the mitophagy‐related genes (six genes; α = 0.0083) and osteogenesis‐related genes (six genes; α = 0.0083) to control the family wise error rate.

## Conflict of Interest

The authors declare no conflict of interest.

## Supporting information

Supplementary

Supplementary
