# Supplementary material for: Contribution of Mitophagy to Cell‐Mediated Mineralization: Revisiting a 50‐Year‐Old Conundrum
Source: Adv Sci (Weinh). 2018 Jul 29;5(10):1800873. doi: 10.1002/advs.201800873 (PMC6193168; doi:10.1002/advs.201800873)
Supplement: Supplementary file 1 — Supplementary [file ADVS-5-1800873-s001.pdf]

Copyright WILEY-VCH Verlag GmbH & Co. KGaA, 69469 Weinheim, Germany, 2018.

ADVANCED SCIENCE
Open Access

Supporting Information

for *Adv. Sci.*, DOI: 10.1002/advs.201800873

Contribution of Mitophagy to Cell-Mediated Mineralization: Revisiting a 50-Year-Old Conundrum

Dan-dan Pei, Jin-long Sun, Chun-hui Zhu, Fu-cong Tian, Kai Jiao, Matthew R. Anderson, Cynthia Yiu, Cui Huang, Chang-xiong Jin, Brian E. Bergeron, Ji-hua Chen,* Franklin R. Tay,* and Li-na Niu*

Supporting Information

Contribution of Mitophagy to Cell-Mediated Mineralization: Revisiting A 50-Year Old Conundrum

Dan-dan Pei[1], Jin-long Sun[1], Chun-hui Zhu, Fu-cong Tian, Kai Jiao, Matthew R. Anderson, Cynthia Yiu, Cui Huang, Chang-xiong Jin, Brian R. Bergeron, Ji-hua Chen, Franklin R. Tay, Li-na Niu**

I. SUPPORTING MATERIALS AND METHODS – Determination of Optimal Concentrations of Mitophagy Inducer and Inhibitors

Mitophagy Induction and Inhibition: Mitophagy was initiated by activation of the mitochondrial permeability transition (MPT) and subsequently, the loss of $\Delta\Psi m$.[1] As a protonophore and uncoupler of oxidative phosphorylation in mitochondria, CCCP is the most commonly used mitophagy inducer in mammalian cells and exerts its effects by reducing $\Delta\Psi$m[2]. By functioning as an inhibitor of the lysosomal proton pump, Baf-A1 inhibits the digestion of damaged mitochondria by lysosomes during the late stage of mitophagy.[3] Being an immunosuppressive undecapeptide, CsA blocks MPT and prevents mitochondrial depolarization.[4]

Cytotoxicity of Mitophagy Inducer/Inhibitor: The osteogenic capacity of the hDPSCs is functionally related to the cytotoxicity of their culturing environment.[5] Hence, stimulators and inhibitors should be used in optimal concentrations that enhance or inhibit mitophagy without adversely affecting cell viability. Unlike most autophagy/mitophagy studies in which the cultured cells were challenged with these reagents for a brief time-period, 2-3 weeks are usually required for the osteogenic lineage-committed hDPSCs to produce macroscopically recognisable extracellular mineral deposits.[6] Accordingly, 3-(4,5-dimethylthiazol-2-yl)-2,5-diphenyltetrazolium bromide (MTT) assay was employed to determine the cytotoxicity of those chemical regents. Differentiated hDPSCs were exposed to different concentrations of CCCP, Baf-A1 or CsA in 96-well plates (N = 6). After 2 or 3 days, 20 μL of MTT was added to each well with a final concentration of 5 mg/mL and incubated at 37 °C for 4 hours. Formazan produced by the cells was dissolved in dimethyl sulfoxide and absorbance was measured at 490 nm using a microplate reader (Synergy HT, BioTek Instruments, Winooski, VT, USA). The optimal concentrations of CCCP, Baf-A1 and CsA determined by MTT assay were further tested for their potential to induce apoptosis.

Effect of Mitophagy Inducer/Inhibitor on Cell Apoptosis: Evaluation was conducted using a Cell Apoptosis Detection Kit (BD Biosciences, San Jose, CA, USA). After culturing for 48 hours with the optimized concentration of CCCP, Baf-A1 or CsA, the cells were washed with cold PBS and re-suspended in binding buffer at 1×10^6 cells/mL. Cells not treated with the chemical reagents were used as control. Then, 100 μL of the solution containing 1×10^5 hDPSCs were transferred and stained with Annexin V- fluorescein isothiocyanate (FITC) and propidium iodide (PI). The cells were sorted with a FACS Calibur flow cytometer (Becton Dickinson, San Jose, CA, USA). Cells that were considered viable were FITC Annexin V- and PI-negative; cells that were in early apoptosis were Annexin V-positive and PI-negative; and cells that were in late apoptosis or necrosis were Annexin V- and PI-positive. Experiments were performed in sextuplicate (N = 6).

Effectiveness of Mitophagy Induction/Inhibition: hDPSCs were treated with the optimized concentration of each mitophagy stimulator/inhibitor and examined by immunofluorescence to determine the effectiveness of these chemical reagents in stimulating/inhibiting mitophagy. Cell culture was performed in 24-well plates with glass cover slips placed at the bottom of each well. The cover slips were retrieved on the 9th day. The attached cells were fixed with 4% formaldehyde, infiltrated with PBS-T (0.1% Triton X-100 in PBS) at 4 °C for 10 min, and blocked with PBS-B solution (1% BSA in PBS solution) for one hour. The cells were then incubated with mouse monoclonal anti-PINK1 antibodies (ab75487, 1:300 in PBS-B; Abcam) or rabbit polyclonal anti-LC3 antibodies (ab128025, 1:500 in PBS-B; Abcam) at 4 °C overnight. This was followed by incubation with goat anti-mouse secondary antibodies conjugated with FITC (ab6785, 1:1000 in PBS-B; Abcam) or goat anti-rabbit secondary antibodies conjugated with cyanine 3 (Cy3, ab98416, Abcam, 1:1000 in PBS-B; Abcam) at 37 °C for 30 min. After rinsing with PBS, the cell-containing cover slips were mounted on glass slides with 4,6-diamidino-2-phenylindole (DAPI)-containing anti-fade mounting medium. Images were captured using confocal laser scanning microscopy (CLSM; model LSM 780, Carl Zeiss Microscopy GmbH, Jena, Germany).

Mitochondria Membrane Potential: Treatment of the differentiated hDPSCs was terminated after 2, 24 and 48 hours by replacing the respective mitophagy stimulator/inhibitor-containing osteogenic differentiation medium (ODM) with pure ODM. Indirect evaluation of $\Delta\Psi m$ was achieved by staining cells with the $\Delta\Psi m$-sensitive dye JC-10 (AAT Bioquest, Sunnyvale, CA, USA). Diluted JC-10 staining solution (JC-10 stock solution in DMSO diluted with Assay Buffer A) was added to the cells and incubated for 30 min at 37 °C in 5% CO2. Assay Buffer

B (50 µL/well) was added to the dye-loaded plate prior to determining the fluorescence intensity at excitation/emission wavelengths of 490/525 nm and 540/590 nm, using the bottom-read mode for ratio analysis. Emission ratio at 525/590 nm was determined from six replicates to represent changes in $\Delta\Psi m$. This is based on the rationale that the monomeric JC-10 dye molecules (green fluorescence, 525 nm) from the cytosol are capable of selectively entering the mitochondria to form dye aggregates with orange fluorescence (590 nm) as the $\Delta\Psi m$ increases.[7] Accordingly, a higher 525/590 ratio indicates a lower $\Delta\Psi m$. The ratio obtained from the mitophagy inducer/inhibitor-treated cells was compared with the control.

References

[1] A. Hamacher-Brady, N. R. Brady, *Cell. Mol. Life Sci.* **2016**, 73, 775.

[2] a) S. R. Yoshii, N. Mizushima, *Int. J. Mol. Sci.* **2017**, 18; b) L. Chen, K. Ma, J. Han, Q. Chen, Y. Zhu, *Methods Enzymol.* **2014**, 547, 39; c) M. Maurolizcano, L. Estebanmartínez, E. Seco, A. Serranopuebla, L. Garcialedo, C. Figueiredopereira, H. L. A. Vieira, P. Boya, *Autophagy* **2015**, 11, 833.

[3] M. Redmann, G. A. Benavides, T. F. Berryhill, W. Y. Wani, X. Ouyang, M. S. Johnson, S. Ravi, S. Barnes, V. M. Darley-Usmar, J. Zhang, *Redox Biol.* **2017**, 11, 73.

[4] V. Leytin, D. J. Allen, A. Mutlu, A. V. Gyulkhandanyan, S. Mykhaylov, J. Freedman, *Lab. Invest.* **2009**, 89, 374.

[5] E. A. Bortoluzzi, L. Niu, C. D. Palani, A. R. Elawady, B. D. Hammond, D. Pei, F. Tian, C. W. Cutler, D. H. Pashley, F. R. Tay, *Dent. Mater.* **2015**, 31, 1510.

[6] L. N. Niu, D. D. Pei, M. Morris, K. Jiao, X. Q. Huang, C. M. Primus, L. F. Susin, B. E. Bergeron, D. H. Pashley, F. R. Tay, *Dent. Mater.* **2016**, 32, 1235.

[7] D. Prantner, D. J. Perkins, W. Lai, M. S. Williams, S. Sharma, K. A. Fitzgerald, S. N. Vogel, *J. Biol. Chem.* **2012**, 287, 39776.

II. SUPPORTING TABLE

Table S1. Primers for Polymerase Chain Reaction

Gene name	Forward primer	Reverse primer
	Mitophagy-related genes	
PINK1	GGACGCTGTTCCTCGTTA	ATCTGCGATCACCAGCCA
PARKIN	AACCGGTACCAGCAGTATGG	TTCGCAGGTGACTTTCCTCT
LC3I	CGTCCTGGACAAGACCAAGT	CTCGTCTTTCTCCTGCTCGT
LC3II	AGCAGCATCCAACCAAAA	CTGTGTCCGTTCACCAACAG
P62	TGCCCAGACTACGACTTGTG	GAGAAGCCCTCAGACAGGTG
LAMP1	GTGTCTGCTGGACGAGAACA	TAGCCTGCGTGACTCCTCTT
LAMP2	AATGCCACTTGCCTTTATGC	CAGTGCCATGGTCTGAAATG
	Mineralization-related genes	
ALP	AAGGACGCTGGGAAATCTG	GGGCATCTCGTTGTCTGAGT
COL1	AGAGGAAGGAAAGCGAGGAG	GGACCAGCAACACCATCTG
OPN	GCCGAGGTGATAGTGTGGTT	ACTCCTCGCTTTCCATGTGT
OCN	CTCACACTCCTCGCCCTATT	CGCCTGGGTCTCTTCACTAC
RUNX-2	GACGAGGCAAGAGTTTCACC	GGTTCCCGAGGTCCATCTAC
OSX	CCCACCTCAGGCTATGCTA	CACTGGGCAGACAGTCAGAA
	Housekeeping gene	
GAPDH	GCACCGTCAAGGCTGAGAAC	TGGTGAAGACGCCAGTGGA

PINK1: Phosphatase-and-tensin homolog-induced putative kinase 1; *LC3* (*LC3A*/*LC3B*): microtubule-associated protein-1 light chain 3; *P62*: Ubiquitin-binding adaptor p62; *LAMP1*: Lysosomal-associated membrane protein 1; *LAMP2*: Lysosomal-associated membrane protein 2; *ALP*: alkaline phosphatase; *COL*-I: type I collagen; *OPN*: osteopontin; *OCN*: osteocalcin; *RUNX2*: runt-related transcription factor 2; *OSX*: osterix; *GAPDH*: glyceraldehyde-3-phosphate dehydrogenase

III. SUPPORTING FIGURES

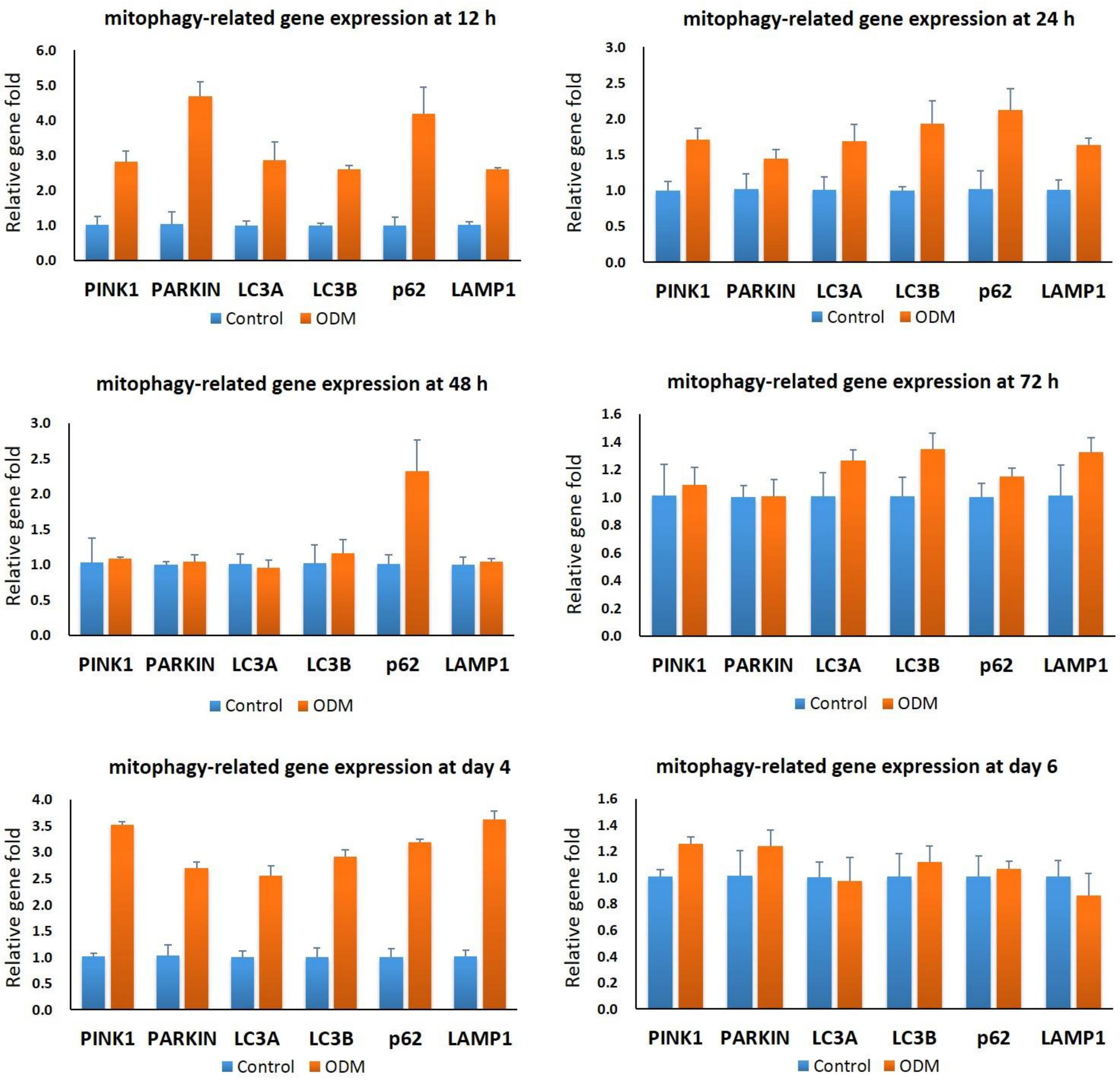

Figure S1. Reverse transcription-polymerase chain reaction of mitophagy-related genes in undifferentiated human dental pulp stem cells (hDPSCs; control) and after the hDPSCs were exposed to osteogenic differentiation medium for different time periods.

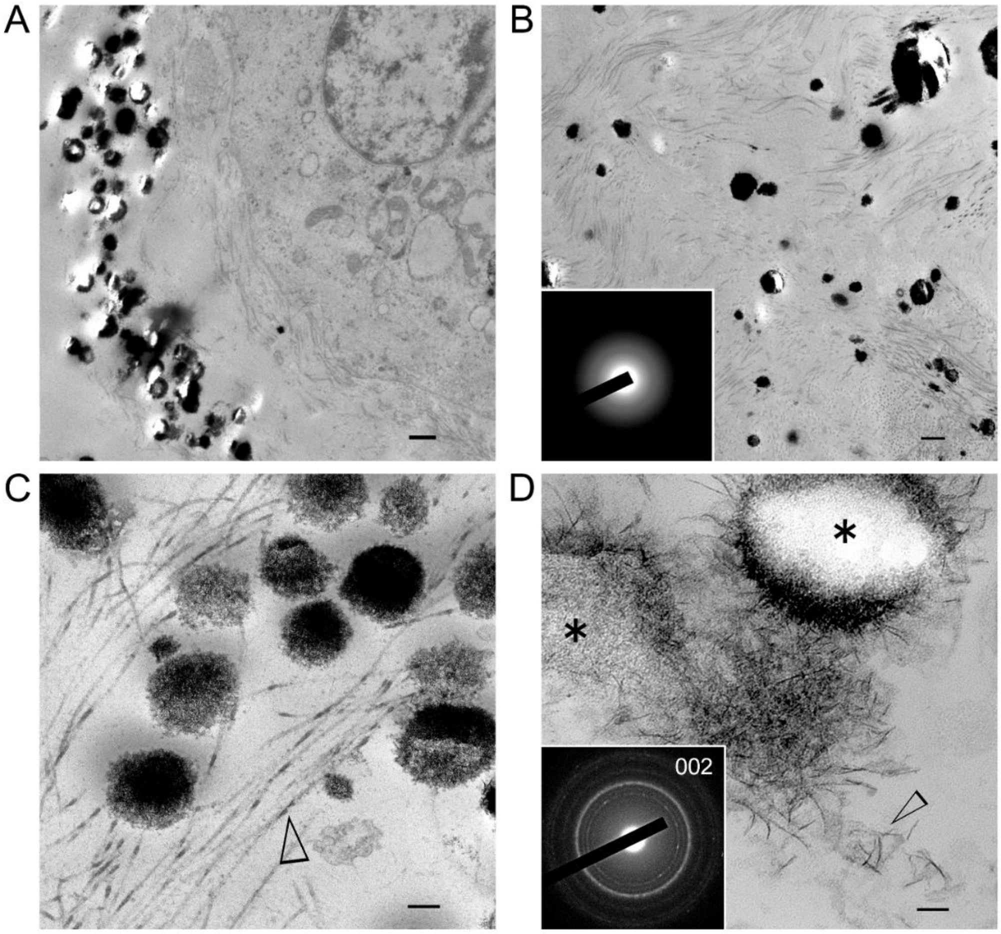

Figure S2. Unstained TEM images of osmicated extracellular matrices produced by differentiated human dental pulp stem cells (hDPSCs) that had been cultured for 48 hours (A–B) and 3 weeks (C–D) in osteogenic differentiation medium. A) Electron-dense mineral granule aggregates adjacent to a cell (bar: 500 nm). B) Mineral granules among unmineralized collagen fibrils were amorphous at this early stage, as shown by selected area electron diffraction (SAED; inset) (bar: 100 nm). C) Extracellular mineral deposits among partially-mineralized collagen fibrils (open arrowhead) in the extracellular matrix (bar: 200 nm). D) High magnification of phase transformation of the ACP deposits (asterisks) into needle-shaped crystallites (open arrowhead). Ring-shaped diffraction patterns produced indicated that the crystallites were apatite (inset).

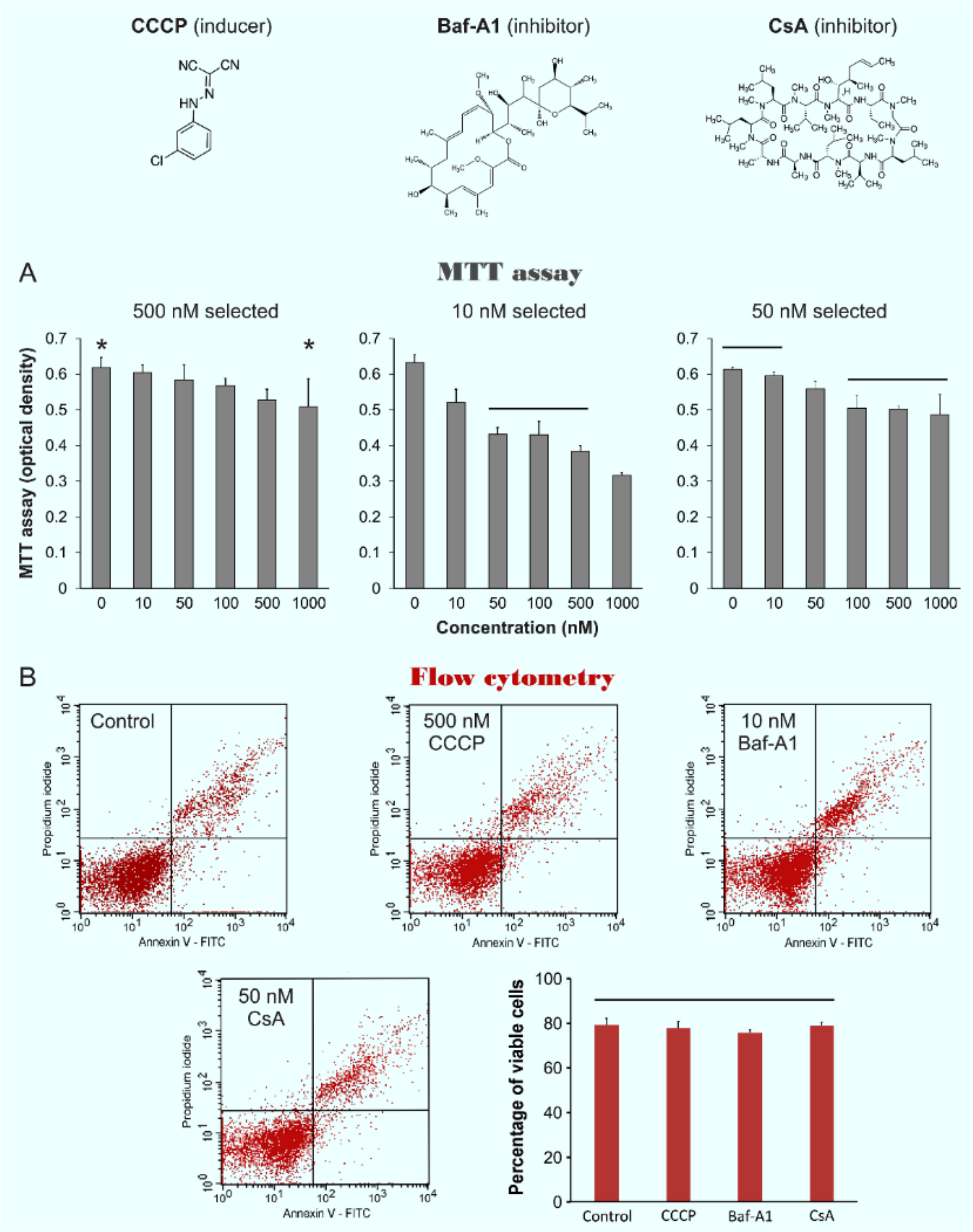

Figure S3. Cytotoxicity evaluations of the viability of differentiated hDPSCs after the cells were exposed to different concentrations of CCCP, Baf-A1 or CsA. (A) MTT assay of succinic dehydrogenase activity. For CCCP, columns designated by asterisks were significantly different ($p < 0.05$). For Baf-A1 and CsA, columns connected by horizontal bars at the same vertical level were not significantly different. Based on the MTT results, 500 nM of CCCP, 10 nM of Baf-A1 and 50 nM of CsA were selected as the respective concentration of the chemical reagent for use in subsequent experiments. (B) The potential of these reagents at their optimized concentration to induce apoptosis/necrosis were evaluated using flow cytometry. Representative 2D dot plots are shown together with a chart comparing the percentage of viable hDPSCs (non-apoptotic, non-necrotic) in each group. Columns designated by the horizontal bar were not significantly different ($p < 0.05$).

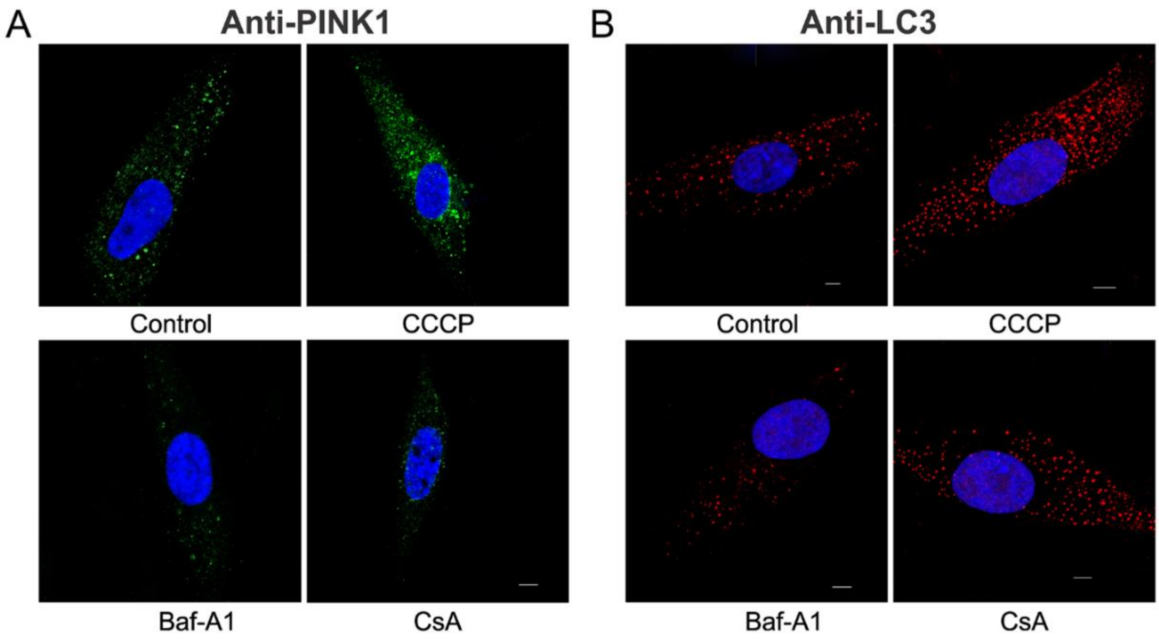

Figure S4. Confocal laser scanning microscopy images of differentiated hDPSCs (control) and hDPSCs treated with CCCP, Baf-A1 or CsA. After the cells were treated for 24 hours, they were labeled with (A) antibodies against PINK1 (green fluorescence) and (B) antibodies against LC3-I/II (red fluorescence).

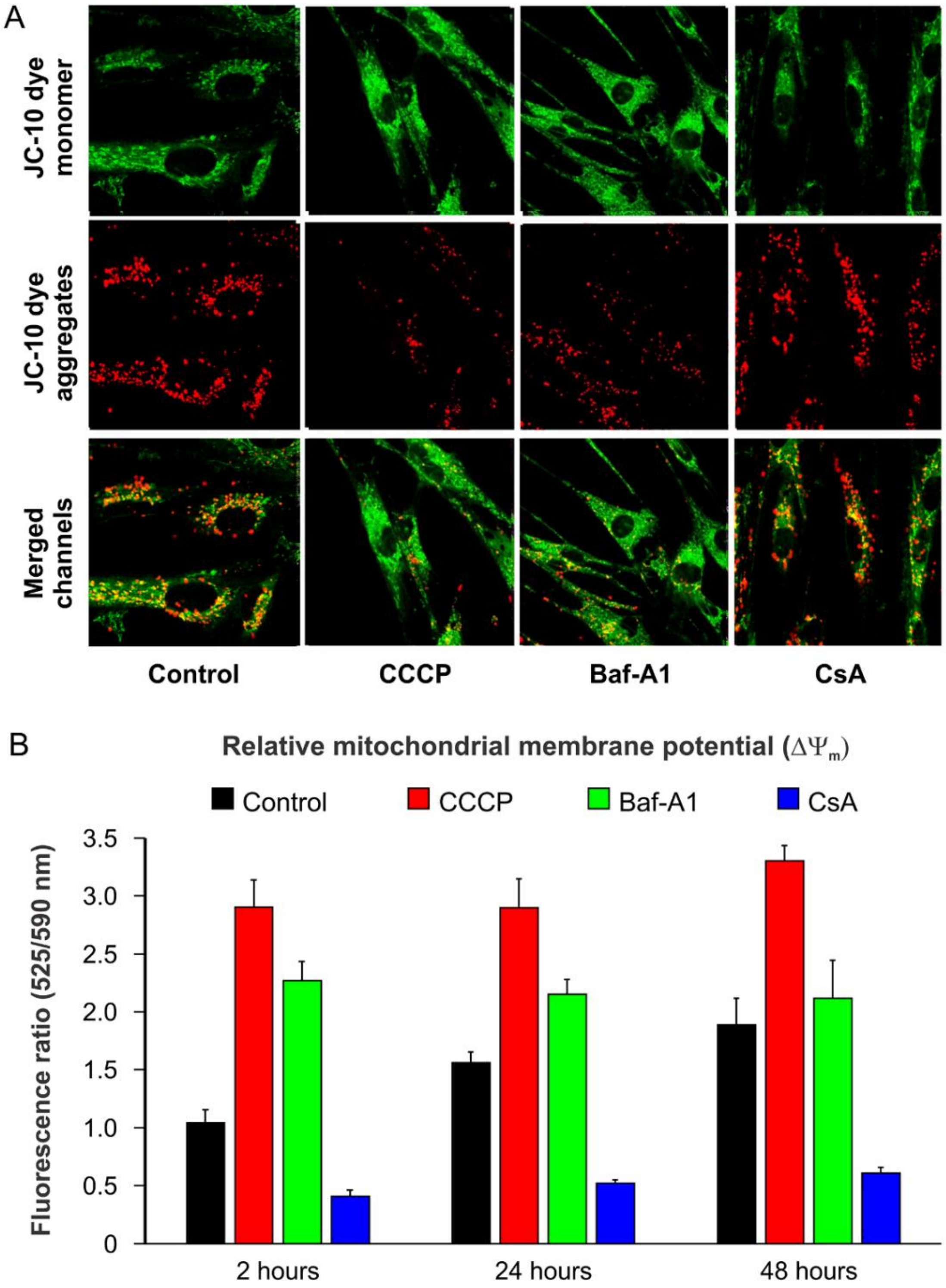

Figure S5. Membrane potential evaluation of differentiated hDPSCs (control) and hDPSCs treated with CCCP, Baf-A1 or CsA. (A) Representative images of reagent-treated hDPSCs that were exposed to the JC-10 dye for analyzing the changes in mitochondrial membrane potential. (B) Changes in mitochondria membrane potential of the reagent-treated and control cells after application of the reagents for 2, 24 and 48 hours. For each group, columns identified with different designators were significantly different ($p < 0.05$).

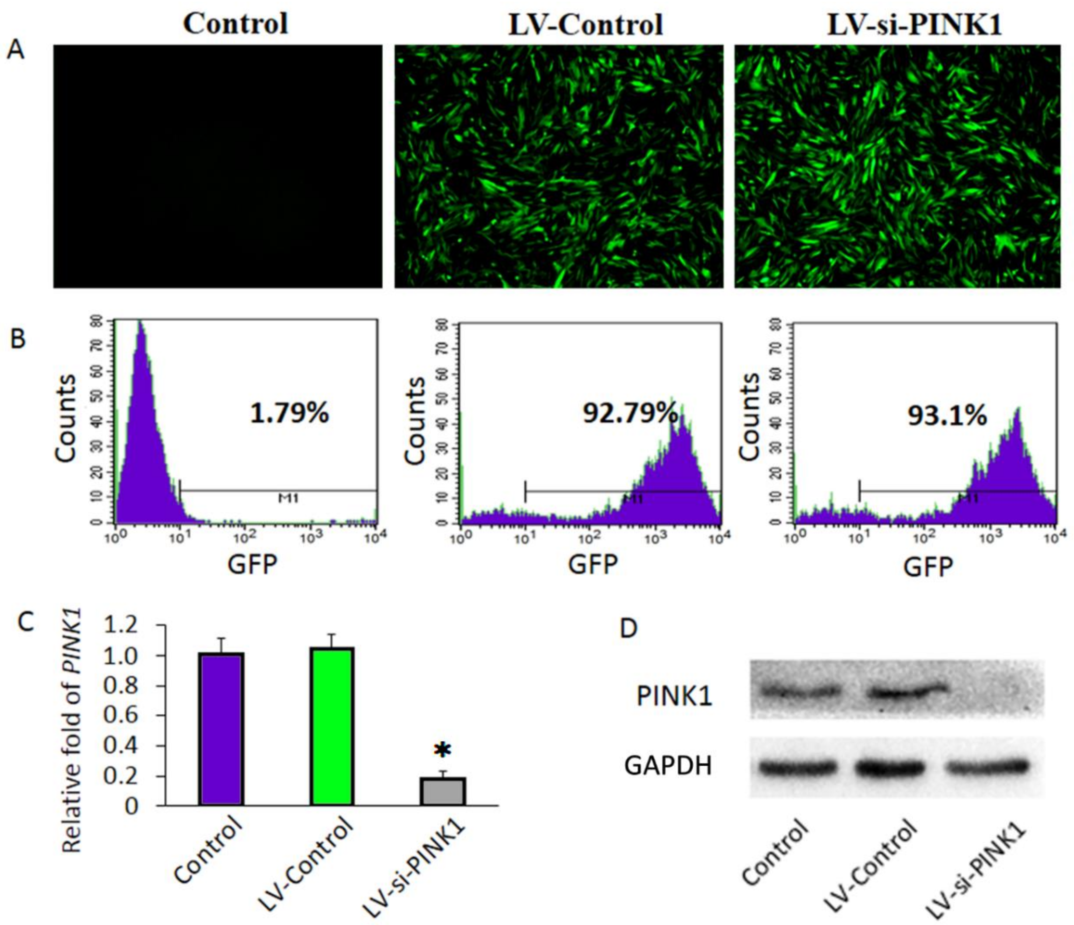

Figure S6. Efficacy of PINK1 silencing by lentivirus transfection. Human dental pulp stem cells transfected with empty lentivirus vector (LV-Control) or PINK1-specific siRNA lentivirus (LV-si-PINK1) for 48 hours. Transfection efficacy was evaluated by (A) fluorescence microscopy and (B) flow cytometry. More than 90% of the transfected cells were GFP-positive. (C) PCR and (D) Western blot were performed to confirm PINK1 silencing effectiveness by lentivirus transfection. GFP: green fluorescent protein. *: $p < 0.001$.

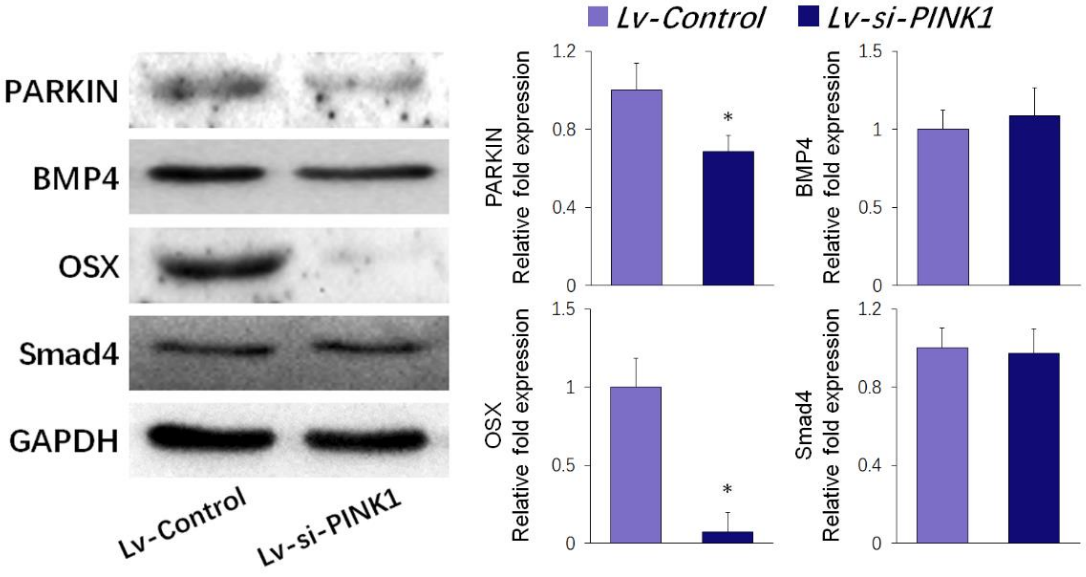

Figure S7. Protein expression of hDPSCs transfected with PINK1-specific siRNA lentivirus. Human dental pulp stem cells transfected with empty lentivirus vector (LV-Control) or PINK1-specific siRNA lentivirus (LV-si-PINK1) for 48 hours. Western blot was performed to evaluate expression of PARKIN, BMP4, OSX and Smad4. *: $p < 0.001$.
